# Active Immunization against Tetanus Attempted upon Man

**Published:** 1918-01

**Authors:** 


**_Active Immunization against Tetanus Attempted upon Man._** By H. Vallée and L. Bazy. Trans. and Abs. from the *Bulletins et Mémoires de la Société de Chirurgie de Paris*, t. XLIII, N° 24, p. 1445. Meeting of June 27, 1917.

The authors, noting the apparently short duration of the immunity conferred by the administration of antitoxic serum against tetanus, have attempted to produce active immunity in man. They have prepared an inert fluid by mixing a toxin, furnished by the Pasteur Institute, with an iodine solution (iodine 1 gr., iodide of potassium 2 gr., distilled water 200 gr.) in the proportion of 2 of toxin to 1 of the iodine solution. The mixture proved non-toxic for animals.

This preparation has been injected in 7 patients in doses of 1 cc. Some had already had serum treatment, which, however, was suspended 5 days previous to the administration of the vaccine. Three doses at intervals of 5 days were injected subcutaneously in the upper part of the affected member.

Ten days later an examination of the blood showed still a high degree of immunity. It was found experimentally that the vaccine rendered a rabbit immune to a very large dose of toxin.

The authors do not think that active immunization should replace the use of antitoxic serum, which, within a limited period of time, they still believe to be the most reliable preventive and curative treatment. They believe, however, that the effects of vaccine are more lasting, and that the serum treatment should be supplemented by a vaccine treatment, administered not earlier than 5 days after the preventive dose of serum.

